# Can larvae of a marine fish adapt to ocean acidification? Evaluating the evolutionary potential of California Grunion (*Leuresthes tenuis*)

**DOI:** 10.1111/eva.12739

**Published:** 2018-12-31

**Authors:** Alexander J. Tasoff, Darren W. Johnson

**Affiliations:** ^1^ Department of Biological Sciences California State University Long Beach California

**Keywords:** animal model, climate change, contemporary evolution, growth, maternal effects, population dynamics, quantitative genetics, recruitment, survival

## Abstract

Ocean acidification can reduce the growth and survival of marine species during their larval stages. However, if populations have the genetic capacity to adapt and increase their tolerance of low pH and high *p*CO_2_ levels, this may offset the harmful effects of ocean acidification. By combining controlled breeding experiments with laboratory manipulations of seawater chemistry, we evaluated genetic variation in tolerance of ocean acidification conditions for a nearshore marine fish, the California Grunion (*Leuresthes tenuis*). Our results indicated that acidification conditions increased overall mortality rates of grunion larvae, but did not have a significant effect on growth. Groups of larvae varied widely with respect to mortality and growth rates in both ambient and acidified conditions. We demonstrate that the potential to evolve in response to ocean acidification is best described by considering additive genetic variation in fitness‐related traits under both ambient and acidified conditions and by evaluating the genetic correlation between traits expressed in these environments. We used a multivariate animal model to estimate additive genetic (co)variance in larval growth and mortality rates under both ambient and acidified conditions (low pH/high *p*CO_2_). Our results suggest appreciable genetic variation in larval mortality rates (*h* ^2^_Ambient_ = 0.120; *h* ^2^_Acidified_ = 0.183; *r_G_* = 0.460), but less genetic variation in growth (*h* ^2^_Ambient_ = 0.092; *h* ^2^_Acidified_ = 0.101; *r_G_* = 0.135). Maternal effects on larval mortality rates accounted for 26%–36% of the variation in phenotypes, but maternal effects accounted for only 8% of the variation in growth. Collectively, our estimates of genetic variation and covariation suggest that populations of California Grunion have the capacity to adapt relatively quickly to long‐term changes in ocean chemistry.

## 1 INTRODUCTION

Anthropogenic carbon dioxide (CO_2_) emissions have substantially changed the chemistry of the ocean (Doney, Fabry, Feely, & Kleypas, 2009; Orr et al., 2005; Sabine et al., 2004). As atmospheric CO_2_ increases, the ocean absorbs excess amounts of atmospheric CO_2_, which causes chemical reactions that emit hydrogen ions and lower the pH of seawater (Zeebe 2012). This chemical process, known as ocean acidification, is expected to proceed at a largely uncontrolled rate. Global ocean pH has decreased by ~0.1 units since the industrial revolution, and may decrease by approximately 0.4 units before the year 2,100 if CO_2_ emissions are not significantly reduced (Bopp et al., 2013; Caldeira & Wickett, 2003). Long‐term ocean acidification will cause widespread effects across marine environments, and it is particularly important to understand how high CO_2_ and low pH levels affect the fitness of marine organisms (Kroeker et al., 2013; Sunday et al., 2014).

It is widely known that ocean acidification can adversely affect the early life stages of marine organisms. For example, bivalve and gastropod larvae developing under low pH/high *p*CO_2_ levels experience lower survival and growth rates than those growing under ambient ocean pH levels (Onitsuka, Kimura, Ono, Takami, & Nojiri, 2014; Talmage & Gobler, 2011). Similarly, coral larvae and juveniles developing under low pH and high *p*CO_2_ levels demonstrate arrested calcification rates caused by the increased dissolution of carbonate ions (Albright, Mason, Miller, & Langdon, 2010). In addition, exposure to low pH seawater can disrupt the energy budgets of some marine species. For example, juvenile crabs in low pH seawater may sustain normal rates of shell calcification by expending a greater amount of energy, which in turn may deteriorate overall body condition (Long, Swiney, Harris, Page, & Foy, 2013).

A growing number of studies suggest that the early life stages of fishes can also be susceptible to the effects of ocean acidification (Baumann, Talmage, & Gobler, 2012; Bignami, Enochs, Manzello, Sponaugle, & Cowen, 2013; Munday et al., 2009; Murray, Malvezzi, Gobler, & Baumann, 2014; Stiasny et al., 2016). In particular, increased CO_2_ in seawater can interfere with the diffusion of metabolic CO_2_ out of the body. Such diffusion is ultimately related to the gradient in *p*CO_2_ between the blood, where concentration of CO_2_ is relatively high, and the external environment, where concentration of CO_2_ is relatively low. Under ocean acidification (OA) conditions, an increase in environmental *p*CO_2_ may result in less of a gradient between blood and the environment. In turn, this may result in an increase in the retention of metabolic CO_2_ and/or prompt a compensatory response that involves the active transport of acid‐base relevant ions and the accumulation of bicarbonate in the body to regulate internal pH (reviewed by Melzner et al., 2009; Heuer & Grosell, 2014). Such regulation may be energetically costly, and long‐term exposure to elevated CO_2_ and low pH levels may disrupt ion regulation. As a consequence, fish developing under OA conditions may experience a variety of harmful effects, including elevated mortality rates, reduced growth rates, and sensory impairment (Allan, Domenici, McCormick, Watson, & Munday, 2013; Bromhead et al., 2015; Frommel et al., 2012; Munday et al., 2009; Nilsson et al., 2012).

**Figure 1:**
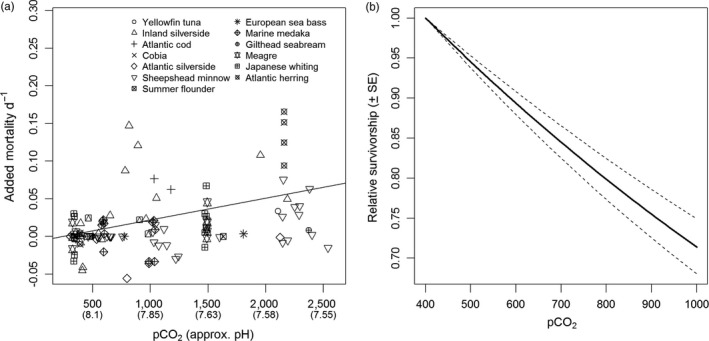
Effects of ocean acidification conditions on the mortality rates of larval fishes. To account for natural differences in overall mortality rates among species and to parse out the component of mortality due to changes in *p*CO_2_ and pH, “added mortality” was calculated by subtracting the average mortality rate at ambient conditions from all replicates in the corresponding experiment. (a) Relationship between *p*CO_2_/pH and added, daily mortality rate for studies of 13 different fishes. (b) Based on the average values in panel (a), a projection of the relationship between *p*CO_2_ and relative survival over a 20‐day period

Although it is clear that changes in seawater chemistry can have harmful effects on the larvae of marine fishes, the overall magnitude of these effects, and their implications for recruitment to populations, may not be appreciated in full detail. To shed some light on this issue and to provide context for the present study, we reviewed and analyzed experimental studies of the effects of changes in *p*CO_2_ and pH on larval mortality (see Supporting Information Appendix S1 for details). Based on the data published thus far (46 experiments total; 13 different species), a unit increase in *p*CO_2_ results in an increase in daily mortality rate of 2.97 × 10^−5^, and a unit decrease in pH results in an increase in daily mortality rate of 0.109, on average (Figure 1a). Species responses’ varied somewhat, but these results suggest that, on average, even small changes in ocean pH can have substantial effects on larval survival and population input. For example, assuming that the effects of reduced pH on mortality persist for at least 20 days (a value near the average study duration), a 0.1 unit decrease in ocean pH may decrease relative survival by 19.5%, on average (Figure 1b). Given that *p*CO_2_ levels are expected to increase by approximately 6 ppm per year over the next century, and ocean pH is anticipated to decrease at a rate of approximately 4.84 × 10^‐3^ units per year (Caldeira & Wickett, 2003), it is possible that many species of fish will experience a long‐term decline in population input. Based on the average rate of OA‐induced mortality, relative survival of larvae could decrease by ~29% by the end of the century (Figure 1b), and such a decrease in larval survival may lead to a substantial, long‐term decline in recruitment to populations (Johnson, Grorud‐Colvert, Sponaugle, & Semmens, 2014).

Such projections assume that current responses to acidification conditions will be the same as future responses. However, if populations can evolve greater tolerance of low pH and high *p*CO_2_, then over several generations the relationship between pH/*p*CO_2_ and mortality may become flatter (i.e., less sensitive), and the decline in relative survivorship with time may slow, or even cease. Because the effects of ocean acidification on survivorship can be substantial, it is important to understand whether populations have the capacity to adapt, and if so, how quickly (Sunday et al., 2014). For populations to evolve in response to acidification, natural selection must act upon phenotypes that reflect tolerance of changes in seawater chemistry. On the timescale of ocean acidification (i.e., decades), evolutionary rates in fish populations will depend primarily on standing genetic variation, rather than new mutations (Gomulkiewicz & Houle, 2009). However, the extent to which tolerance of ocean acidification conditions is heritable and passes between generations in fish populations is largely unknown (but see Malvezzi et al., 2015 for a detailed study of genetic variation in survival times under high CO_2_/low pH conditions).

Tolerance of ocean acidification conditions can be measured by comparing the performance of larvae (e.g., survival, growth) in both ambient and acidified seawater. If differences in performance are small between these treatments, it indicates that larvae are tolerant of ocean acidification conditions. In contrast, if the differences in performance are large, then it indicates that larvae are sensitive to changes in pH and/or *p*CO_2_. Although individuals are either alive or dead, average mortality rates can be measured for groups (e.g., families of larvae), and multiple measurements can be made for the same group. Measures of OA tolerance (via differences in mortality rates) can therefore be analyzed as quantitative traits. After all, traits such as these are likely influenced by a large number of genes (each with a small additive effect), as well as environmental sources of variation (Besnier et al., 2015).

We used quantitative genetic analyses to assess whether larvae of a coastal marine fish may have the potential to adapt to become more tolerant of ocean acidification. Specifically, we combined a cross‐breeding experiment (which produced groups of larvae of varying degrees of relatedness) with an experimental manipulation of pH and *p*CO_2_ under laboratory conditions. This approach allowed us to compare the overall sensitivity of this population to ocean acidification conditions. We were also able to measure the genetic capacity of this population to evolve in response to ocean acidification. This was accomplished by estimating multiple components of genetic variation, including both the heritabilities of mortality rate and growth under ambient and acidified conditions and the genetic correlations between traits expressed in different environments (i.e., under ambient and acidified conditions). Heritability describes the proportion of total phenotypic variation that is due to additive genetic variation and thus describes how quickly a population can respond to a unit of selection (Falconer & Mackay, 1996). In the context of ocean acidification, heritabilities of traits under ambient conditions provide a measure of how quickly these traits may evolve if selected upon under *current *conditions. Quantifying how a population will evolve *as the environment transitions* from ambient conditions to an acidified state will also require an estimate of genetic correlations between traits expressed in current and future conditions (e.g., mortality rate under ambient and acidified treatments). Genetic correlations can measure the degree to which expression of traits in ambient and acidified conditions is affected by the same set of alleles. Genetic correlations thus provide a measure of whether evolved responses to changes in seawater chemistry in the current generation can also make the population more tolerant of future, acidified conditions. Such information is critical for understanding populations’ long‐term responses to ocean acidification.

## 2 METHODS

### 2.1 Study organism

Our study examined the adaptive capacity of California Grunion (*Leuresthes tenuis; *Ayers), a coastal forage fish within the family of New World silversides (Atherinopsidae). This species is found throughout the nearshore waters of Southern California, and adults can grow to 14–19 cm total length. Grunion are renowned for their habit of spawning on sandy beaches. During spawning events (a.k.a. grunion runs), females deposit their eggs below the surface of the sand, while males surround the females and fertilize the newly released eggs (Smyder, Martin, & Gatten, 2002). Grunion embryos develop for about 2 weeks and typically hatch during the next springtide (Griem & Martin, 2000). Spawning grunion can be caught by hand during grunion runs and can be artificially spawned in controlled breeding experiments. In such experiments, the pedigree of offspring is known, and there are clear expectations for degree of phenotypic similarity among relatives. In addition, grunion share many life‐history traits with other nearshore fishes. These traits include iteroparous spawning behavior; an extended pelagic larval stage; and continual genetic exchange between large populations (Gaida et al., 2003; Houde & Zastrow, 1993; Johnson et al., 2009). Thus, grunion may serve as a convenient model species for studying the potential for marine fish larvae to adapt to changes in seawater chemistry.

### 2.2 Breeding experiment

To estimate the genetic variance underlying tolerance of OA conditions, we used a crossed breeding design to produce offspring of various degrees of relatedness and then compared the performance of those larval offspring under both ambient and OA conditions in laboratory experiments. To produce offspring for these experiments (details of which are described in the next subsection), adult grunion were hand‐caught and strip‐spawned into small plastic containers. Eggs of each female were divided into three equal groups and fertilized by three different males. The sperm from each male was used to fertilize eggs from three different females. Each group was thus a full sibling family, and larvae from separate groups could be paternal half‐sibs, maternal half‐sibs, or unrelated. During spawning events, the design of three males crossed with three females in all combinations was usually repeated twice to produce 18 families in total, though in a few cases when spawning adults were scarce, we used a single set of three males and three females. Fertilized eggs were placed into separate 475‐ml containers, covered with 200 ml of moist beach sand, and stored at room temperature (20–21°C) for 14 days to allow eggs to undergo normal development (Ehrlich & Farris, 1972; Smyder et al., 2002). To prevent egg desiccation, each container was sprayed with seawater every other day until the sand had a moisture content between 60% and 90%. Adults were collected and spawned in the 2015, 2016, and 2017 spawning seasons (March through August) at Seal Beach, California (33.740°N, 118.114°W). Grunion spawn every 2 weeks during the spawning season, and we used offspring produced in 16 different spawning events. These events were treated as replicate, temporal blocks in our laboratory experiments (6 blocks in year 1, 6 blocks in year 2, and 4 blocks in year 3).

### 2.3 Seawater chemistry manipulation and laboratory experiment

In this experiment, larvae from each family were reared under ambient conditions and OA conditions (low pH and high *p*CO2). We used one recirculating seawater system to simulate OA conditions (*p*CO_2_ ~ 1,630 μatm; pH_NBS_ ~ 7.6), and another system to simulate ambient conditions (*p*CO_2_ ~ 650 μatm; pH_NBS_ ~ 8.0). The ambient treatment represented pH and *p*CO_2 _levels within nearshore waters of the Southern California Bight (Davidson 2015, Jones, Sweet, Brzezinski, McNair & Passow, 2016). The *p*CO_2_ and pH in our OA treatment were chosen based on levels that are likely to occur within Southern California in the next 100 years (Gruber et al., 2012; Turi, Lachkar, Gruber, & Munnich, 2016). Each system had 12 larval rearing tanks, and each tank was connected to a larger sump in which water was filtered and aerated before recirculating within the system (Supporting Information Appendix S2). Rearing tanks were 6.6 L in volume and fitted with acrylic tops to minimize evaporation. In the OA treatment, both air and CO_2_ were bubbled into the system, and gas delivery was regulated by an automated pH‐stat system (Pinpoint‐pH, American Marine Inc., Ridgefield, CT) which monitored seawater pH and controlled the delivery of gases into the system. Systems were supplied with seawater that was collected from our study area (Seal Beach, CA), and then circulated through a 10‐micron filter and an ultraviolet sterilizer (Turbotwist 6X, Coralife, Franklin, WI) before being used in the experiments. Our larval rearing experiment was replicated in blocks, and between blocks, each seawater system received partial to full water changes (80%–100% volume) to avoid waste buildup. In addition, between each block, the experimental treatment assigned to each seawater system was assigned at random. This procedure helped guard against any unforeseen effects associated with the location of tanks within the laboratory. Water was replenished with freshly collected seawater, and water was replaced at various times during the spawning season and study years. This introduced appreciable variation in seawater characteristics such as alkalinity, but this variation reflects the natural variation in seawater chemistry within nearshore environments of Southern California (Davidson 2015, Jones et al., 2016).

Seawater chemistry was monitored throughout the experiment. We used a multiparameter probe (ProDSS, YSI Inc., Yellow Springs, OH, USA) to record the pH, salinity, and temperature (°C) within each system every 1–3 days. Probes were routinely calibrated with buffers certified by the National Institute of Standards and Technology (YSI Inc.), and all measurements were recorded to the nearest 0.01 units. In addition, we took water samples every 1–2 weeks to calculate the total alkalinity (TA) using general acid titration (Clesceri, Greenberg, & Eaton, 1999). Titrations were performed by Physis Environmental Laboratories (Long Beach, CA), and each set of measurements was validated with a procedural control. When validated with certified reference material (courtesy of A. Dickson, Scripps Institution of Oceanography), estimates of total alkalinity were within 0.3%–1.8% of values reported for the reference material, with a mean percentage error of 0.9%. Values of *p*CO_2_ were then quantified based on the collected data and carbonic acid dissociation constants for seawater using CO2SYS (http://cdiac.ess-dive.lbl.gov; Pierrot & Wallace, 2006). Our seawater chemistry measurements are summarized in Supporting Information Appendix S2.

Following 14‐days of incubation, eggs were cued to hatch by agitating the eggs with seawater for 1 min (Griem & Martin, 2000; Martin, Moravek, & Walker, 2011). Hatched larvae from each family were partitioned between the two OA treatments in groups of 50 larvae per rearing tank. Although each spawning event consisted of 1–2 sets of three males crossed with three females and could thus produce between 9 and 18 families of larvae, we could rear a maximum of 12 groups of larvae within the laboratory at a time. Within each block (*n*_blocks_ = 16), we chose a random subset of families to rear within the experiment. Additionally, many families were further separated into multiple groups to provide replicate measures of the same families in different containers. This allowed us to evaluate variation among tanks, including variation that may have been caused by unforeseen differences in laboratory conditions such as lighting and water flow. Thirty‐one families were replicated, and these families had between two and six replicates. Larvae remained in the rearing tanks for 14 days under a seawater flow regime of 10 ml/s and a 11:13‐hr light–dark cycle. Two days after hatching, larvae began a daily diet of 160 brine shrimp nauplii (*Artemia *sp.) per individual, a feeding level known to produce natural growth rates in laboratory reared grunion larvae (May, 1971). The breeding design yielded a total of 153 replicates including 76 sets of paternal half‐sibling families, 67 sets of maternal half‐sibling families, and 31 sets of full siblings with duplicate families.

To measure mortality for each family and OA treatment, rearing tanks were siphoned daily to remove and enumerate deceased individuals. Daily mortality rates were calculated as the negative of the slope of linear regressions that described changes in the natural logarithm of surviving larvae over time. To calculate growth rates, 10 larvae from each family and pH level were sampled at the beginning of the experiment and the end of the experiment (14 day). Sampled larvae were euthanized within a solution of tricaine methanesulfonate (MS‐222) and photographed under a dissecting microscope. Growth was measured as the change in mean standard length throughout the experiment.

### 2.4 Quantitative genetic analyses

To analyze variation in mortality and growth under both ambient and acidified conditions, we used a multivariate animal model: a type of mixed‐effects model that can estimate additive genetic variation by comparing the phenotypic similarities of relatives with known degrees of relatedness (Johnson, Christie, & Moye, 2010; Kruuk 2004; Wilson et al. 2010). For example, if additive genetic variance (*a*) is a component of phenotypic variation, then full siblings (which share half their genome, on average) will be similar by a factor of 0.5*a*, and half‐siblings (which share a quarter of their genome, on average) will be similar by a factor of 0.25*a*. The observed degree of phenotypic similarity and the known genetic relationships for various types of relatives can be used to estimate additive genetic variances and covariances. In our analyses, the four measurements of larval performance (mortality and growth under both ambient and acidified conditions) were treated as four traits that could potentially covary. We partitioned (co)variation in larval traits into four causal components: additive genetic effects, maternal effects, variance among blocks, and residual variation. Each (co)variance component was treated as a random effect, and the phenotypic variance–covariance matrix (**P**) was modeled as a combination of four variance–covariance matrices:

P=G+M+B+R

where **G** is the variance–covariance matrix of additive genetic effects, **M** represents maternal effects, **B** represents variance among blocks, and **R** represents residual variation (in this case variation among replicate rearing tanks). The diagonal elements of each matrix contained components of variance for larval mortality under ambient and acidified conditions and larval growth under ambient and acidified conditions. The off‐diagonal elements within each matrix represented the covariances among larval traits. The only fixed effects were the estimated mean values for each of the four traits. Models were fitted using Monte Carlo Markov chain (MCMC) methods found in the R software package *MCMCglmm* (Hadfield, 2010). To construct a weakly informative prior distribution for the composition of **P**, we set the modes of the component distributions such that each component accounted for ¼ of the total phenotypic variation, but we parameterized these distributions such that they were very wide and flat. Specifically, we used an inverse Wishart distribution for each (co)variance component. The modes of the variance terms were ¼ of the observed variance, the modal covariances were zero, and the degree‐of‐belief parameter was 0.001—a value that is extremely low and minimizes the influence of the priors (Hadfield, 2010). The *glmm* ran for 150,000 iterations, with a burn‐in interval of 1,000 and a thin period of 80. Convergence for each model was assessed by verifying that autocorrelation values for MCMC‐sampled parameters were approximately zero, that the posterior distributions were smooth and unimodal, and that all estimates passed the diagnostic tests of Heidelberger and Welch (1983).

Our analyses focused on estimating additive genetic variances and covariances of larval traits under ambient and acidified conditions because it is the combination of these properties that indicates whether larval traits have the genetic capacity to evolve in response to ocean acidification. Expressing additive genetic variance as a proportion of the total yields a measure of heritability. To keep other values comparable, all variance components were expressed as proportions of the total variance. We also estimated genetic and maternal effect correlations. These quantities express the amount of genetic and maternal variance that is shared between traits. Genetic correlations between two traits were calculated as

$$r_{G}\;\left(x,\;y\right)\;=\;\frac{{\text{Cov}}_{A}\;\left(x,\;y\right)}{\sqrt{V_{A}\left(x\right)\;\times \;V_{A}\left(y\right)}}$$

where Cov*_A_* is additive genetic covariance, *V_A_* is additive genetic variance. A similar formula was used to calculate maternal effect correlations (*r_M_*) based on maternal variances and covariances (elements of the **M** matrix estimated by the multivariate animal model).

## 3 RESULTS

**Figure 2:**
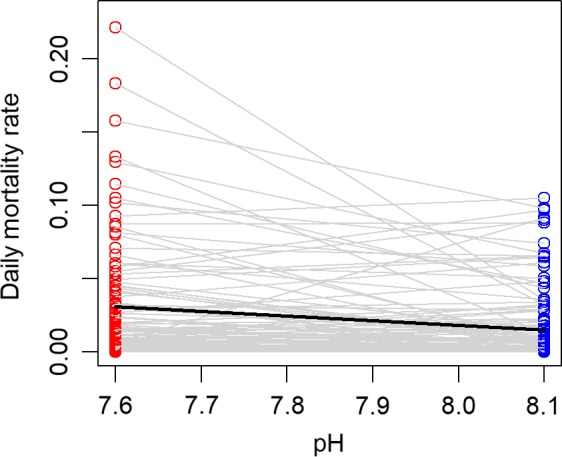
Daily mortality rates for replicate groups of larval grunion that were reared under both low and ambient pH conditions. Gray lines connect values for each replicate. Experiments ran for 14 days, and data are presented for 153 replicates

**Figure 3:**
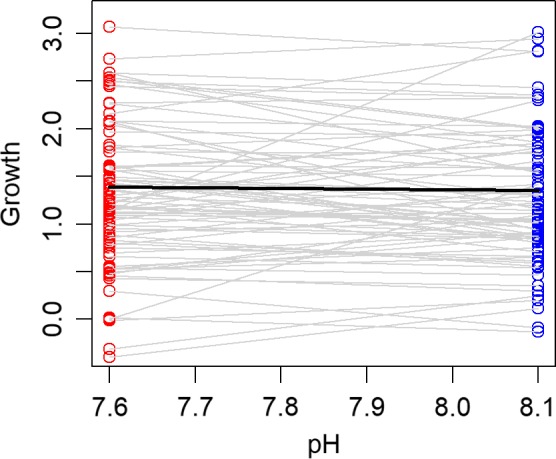
Mean values of growth for replicate groups of larval grunion that were reared under both low and ambient pH conditions. Growth was measured as the difference in average standard length (in mm) between day 14 and day 0 of the experiment. Gray lines connect values for each replicate. Experiments ran for 14 days, and data are presented for 153 replicates

Overall, the mortality rates of larvae in the low‐pH treatments were significantly higher than those in the ambient pH treatments (average difference in daily mortality rates = 0.0126, paired *t* test using blocks as replicates: *t = *2.36, *df* = 15, *p* = 0.032). When these values are expressed as survivorship over the 14‐day duration of the experiment, they indicate that ocean acidification conditions decreased survivorship of larvae by 16% on average. Despite the overall increase in mortality with low pH, different groups of larvae (each of which was a full sibling family) varied widely with respect to differences in mortality between the pH treatments. Some groups exhibited high tolerances (little difference in mortality rates between low and ambient pH), and others exhibited a high degree of sensitivity to the effects of pH and *p*CO_2_ (Figure 2). In addition, there was greater variation in mortality rates for the low pH, high *p*CO_2_ treatment. In contrast, ocean acidification conditions had little to no effect on the growth rate of larvae. Growth rates tended to be slightly higher in reduced pH conditions, but the overall difference was statistically indistinguishable from zero (average difference in growth = 0.017, paired *t* test using blocks as replicates: *t* = 0.283, *df* = 15, *p* = 0.781). Families varied substantially with respect to differences in growth rates between the pH treatments (Figure 3).

**Figure 4:**
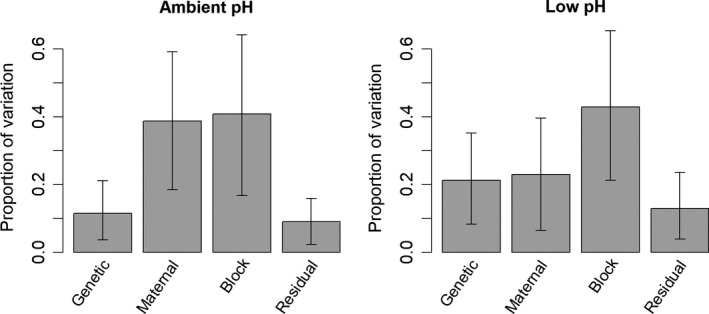
Estimated components of variation in daily mortality rates of larvae. Bars indicate the estimated proportion, and error bars represent 95% credible intervals

Quantitative genetic analyses revealed an appreciable amount of genetic variation in larval mortality rates. Under ambient conditions, additive genetic variation accounted for 12.0% of the variation (heritability = 0.120; 95% credible interval 0.038–0.228). Maternal effects accounted for a considerable proportion of the variation (0.363; 95% CI: 0.159–0.600), block effects were the largest component (0.426; 95% CI: 0.167–0.690), and residual effects accounted for the remainder (0.091; 95% CI: 0.027–0.167; Figure 4). Under acidified conditions, additive genetic variation accounted for slightly more of the variation in larval mortality rates (heritability = 0.183; 95% credible interval 0.050–0.330), maternal effect variation was smaller (0.265; 95% CI: 0.076–0.453), variation among blocks was largest (0.423; 95% CI: 0.163–0.678), and residual variation was the smallest component (0.129; 95% CI: 0.029–0.229; Figure 4).

There was a moderate genetic correlation between larval mortality rates under ambient and acidified conditions (*r_G_* = 0.460, CI: 0.023–0.842). This value suggests some genetic commonalities between mortality rates in the two environments (e.g., some of the alleles that confer a survival benefit under ambient conditions also confer a survival benefit under high *p*CO_2_ and low pH conditions). However, the genetic correlation was much lower than 1, suggesting a genotype by environment interaction in which genotypes that had low mortality rates under ambient conditions did not necessarily have low mortality rates under acidified conditions. In addition, maternal effects on larval mortality rates tended to be shared across the *p*CO_2_ and pH environments. Mothers whose offspring exhibited low mortality rates in ambient conditions also exhibited low mortality under acidified conditions, leading to a strong correlation of maternal effects (*r_M_* = 0.820, 95% CI = 0.631–0.949).

**Figure 5:**
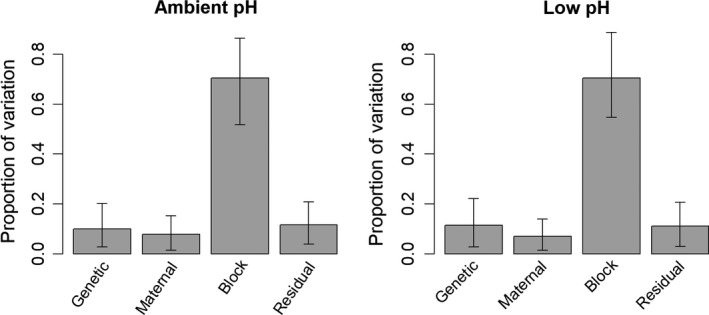
Estimated components of variation in growth of larvae. Bars indicate the estimated proportion, and error bars represent 95% credible intervals

Genetic variation was a smaller component of the total variation in growth rates. Under ambient conditions, additive genetic variance accounted for 9.2% of the variation (heritability = 0.092, 95% CI = 0.023–0.176). Maternal effects were a minor component of variation (0.080; 95% CI = 0.018–0.159), variation among blocks was large (0.728; 95% CI = 0.538–0.885), and residual variation was a small component (0.100; 95% CI = 0.032–0.181; Figure 5). Under acidified conditions, additive genetic variance accounted for 10.1% of the variation (heritability = 0.101, 95% CI = 0.029–0.200). Maternal effects were a similarly small component (0.080; 95% CI = 0.017–0.162), variation among blocks was large (0.718; 95% CI = 0.554–0.901), and residual variation was relatively small (0.096; 95% CI = 0.027–0.173; Figure 5).

There was a small genetic correlation between larval growth under ambient and acidified conditions (*r_G_* = 0.135; CI: −0.345–0.575) indicating a genotype by environment interaction, and little‐to‐no genetic correlation between mortality and growth under both ambient conditions (*r_G_* = 0.041; CI: −0.479–0.510) and acidified conditions (*r_G_* = 0.048; CI: −0.475–0.524). Maternal effects on growth appeared to be shared between environments to a moderate extent. Mothers whose offspring grew quickly in ambient conditions also tended to have offspring that grew quickly in acidified conditions (*r_M_* = 0.337, 95% CI = −0.094–0.744). Finally, there was some evidence of a shared maternal effect between mortality rates and growth. Mothers whose offspring grew quickly also tended to have higher mortality rates, resulting in a positive correlation of maternal effects for both ambient conditions (*r_M_* = 0.320, 95% CI = −0.183–0.741) and acidified conditions (*r_M_* = 0.283, 95% CI = −0.200–0.723).

## 4 DISCUSSION

Our results indicate that changes in seawater chemistry can affect the mortality of larvae and therefore the overall fitness of California Grunion. When comparing survivorship in the ambient and acidified treatments, ocean acidification conditions decreased 14‐day survivorship of larvae by 16%. Sensitivity of grunion larvae to OA conditions was lower than average for marine fish larvae, but well within the range of values reported thus far. Grunion mortality rate increased by 0.066/day per unit decrease in pH (NBS scale), and although the average rate of increase was 0.109/day, the sensitivity of California Grunion larvae was ranked 6th highest out of the 14 species in our review (Supporting Information Appendix S1). Marine fishes appear to vary substantially with respect to their sensitivity to changes in seawater chemistry. For example, Baumann et al. (2012) reported that early spring survivorship of Inland silverside larvae (*Menidia beryllina*) developing for 7 days at low pH and high *p*CO_2_ (~7.78/1,000 ppm) was 26% of what it was under ambient conditions (~8.15/400 ppm). Similarly, Stiasny et al. (2013) reported that relative survivorship of Atlantic cod larvae (*Gadus morhua*) developing for 23.5 days at low pH and high *p*CO_2_ (~7.68/1,106 μatm) was ~30% of what it was at under ambient conditions (~8.04/465 μatm). In contrast, other species appear to be less sensitive. For example, survival of summer flounder (*Paralichthys dentatus*) larvae over a 28‐day period was 91% of the control values when larvae developed in seawater of pH 7.5 and *p*CO_2_ of 1,800 μatm, and equivalent to control values when developing in seawater of pH 7.06 and *p*CO_2_ of 4,700 μatm (Chambers et al., 2014). Similarly, Pope et al. (2014) found that larvae of European sea bass (*Dicentrarchus labrax*) experienced slightly lower mortality rates under acidified conditions. Reasons for variation in pH tolerance among species are not entirely clear, but it is possible that tolerance may be related to larval size. The two variables are modestly correlated (*r* = 0.294), but relatively few studies of pH tolerance have been published thus far, and the statistical evidence of this relationship is weak (*df* = 12, *p* = 0.30; Supporting Information Appendix S1).

Changes in seawater chemistry had little to no effect on the overall growth rate of grunion larvae. Although these results were somewhat surprising, effects of reduced pH and elevated *p*CO_2 _on growth of larval fishes appear to be variable. Some studies have found that OA conditions negatively affect larval growth (Baumann et al., 2012; Frommel et al., 2016; Miller, Watson, Donelson, Mccormick, & Munday, 2012), whereas other studies have found no effect (Bignami et al., 2013; DePasquale, Baumann, & Gobler, 2015; Franke & Clemmesen, 2011; Hurst, Fernandez, & Mathis, 2013), or even a positive effect (Munday et al., 2009; Rossi et al., 2015). Species may vary with respect to their sensitivity to OA conditions, but it is also possible that the effects of pH and *p*CO_2 _on growth also depend on other conditions, such as food availability. Exposure to reduced pH seawater may increase the energetic costs to maintain homeostasis, particularly internal acid‐base regulation (Stumpp, Trübenbach, Brennecke, Hu, & Melzner, 2012; Tseng et al., 2013). This energetic cost may result in reduced growth, but it has been demonstrated that some larval organisms may sustain adequate growth rates under ocean acidification when provided sufficient amounts of food (Pan, Applebaum, & Manahan, 2015). During this study, grunion larvae were fed at rates known to produce normal growth rates under laboratory conditions (May, 1971). It is possible that the energy ingested by larvae was enough to compensate for the high energetic demands for homeostasis and growth under low pH levels. Future studies should concentrate on understanding how vital rates of larvae are affected by the interactive effects of OA conditions and other factors such as food availability, temperature, and dissolved oxygen, since these factors will also be affected by climate change (DePasquale et al., 2015; Gobler, DePasquale, Griffith, & Baumann, 2014; also see reviews by Pörtner, Langenbuch, & Michaelidis, 2005; Wallace, Baumann, Grear, Aller, & Gobler, 2014). In addition, our study was relatively short term (14 day) and it is possible that OA conditions affect growth later in life and/or OA effects are compensated for later in life. Future studies that examine responses over longer time periods will be valuable.

In this study, groups of larvae demonstrated broad variation in mortality and growth under both ambient and acidified conditions. This phenotypic variation was composed of genetic, maternal, and environmental components. The environmental component was the largest and most apparent as variation among replicate blocks. This variation may reflect seasonal and yearly variation in water conditions, and/or the conditions of the parents. For example, water temperature and food availability are dynamic in nearshore waters, and the females used in this experiment would have developed their eggs under different sets of ocean conditions. Additionally, average size and condition of spawning females varied somewhat among spawning events (D. Johnson, *unpublished data*). It is possible that some of the among‐block variation represents transgenerational effects (Murray et al., 2014). Transgenerational effects and among‐block variation in ocean conditions were not the focus of our study, but it is important to evaluate heritability and genetic correlations in light of these other sources of phenotypic variation (Wilson 2008). Maternal effects on larval mortality rates in both ambient and OA conditions were a substantial source of variation, and these results are consistent with many other studies suggesting strong maternal effects on mortality rates of fishes (see reviews by Chambers & Leggett, 1996, Green, 2008, Hixon, Johnson, & Sogard, 2014). Heritability of larval mortality rates was low but appreciable under ambient conditions (*h* ^2^ = 0.120) and slightly higher under acidified conditions (*h* ^2^ = 0.183). We know of only one other study that has measured heritability of survival responding to pH in a marine fish. Malvezzi et al. (2015) studied Atlantic silversides (*Menidia menidia*) and estimated heritability of individual survival times under high CO_2_ conditions to be 0.196. This trait is similar to larval mortality rates, and the results are encouraging for the long‐term prospects of fish populations facing changes in seawater conditions.

There have now been a number of studies that have used quantitative genetics to evaluate the potential for marine species to adapt to climate change (Foo, Dworjanyn, Poore, & Byrne, 2012, 2016; Kelly, Padilla‐Gamiño, & Hofmann, 2013; Shama, Strobel, Mark, & Wegner, 2014; Munday, Donelson, & Domingos, 2017; Sunday, Crim, Harley, & Hart, 2011; Welch & Munday 2017). Most of these studies have focused on measuring heritability of traits under projected, future seawater conditions. For example, heritable variation in the size of larvae under acidified conditions has been demonstrated for several marine invertebrates (Foo et al., 2012; Kelly et al., 2013; Sunday et al., 2011), and heritable variation in survival under acidified conditions has been demonstrated for a marine fish (Malvezzi et al., 2015). An emerging trend is that there appears to be an appreciable amount of standing genetic variation in traits related to performance and survival under future ocean conditions, including increased temperature, increased CO_2_, and reduced pH.

Such results suggest that contemporary evolution may play a role in marine species’ long‐term responses to climate change. However, genetic variation in performance under acidified (future) conditions provides only a partial evaluation of a population's potential to evolve. Measures of genetic variation and heritability should be interpreted in the context of selection, and in the case of ocean acidification, there is no straightforward measure of selection under future conditions. Projections of evolutionary responses (e.g., using the Breeder's equation, $\Delta \overset{¯}{z}\;=\;h^{2}S$, to project the change in mean phenotype, $\Delta \overset{¯}{z}$, from measures of heritability, *h* ^2^, and selection within a generation, *S*) can be misleading if one considers heritability and selection on traits under acidified conditions only. Ocean acidification is not an instantaneous process, and the environmental transition should be taken into account when considering both trait expression and selection. Because ocean acidification is a relatively slow transition from ambient to more acidified conditions, populations will experience a gradual shift in natural selection across multiple generations. A population's capacity to evolve in response to such selection will be determined mainly by heritability under ambient conditions and by the genetic correlations between the trait(s) expressed under ambient and acidified conditions. If the genetic correlations are positive, then as the environment transitions toward a more acidified state, selection in the current generation may cause a correlated genetic response which may “prime” the population to be more tolerant of future seawater conditions. For example, in our study, the genetic variance–covariance matrix (**G**) for larval mortality rates was estimated to be

$$\begin{array}{cc} \text{2.48}\;\times \;{\text{10}}^{\;\text{- 4}} & \text{2.10}\;\times \;{\text{10}}^{\;\text{- 4}}\\ \text{2.10}\;\times \;{\text{10}}^{\;\text{- 4}} & \text{7.23}\;\times \;{\text{10}}^{\;\text{- 4}} \end{array},$$

where G*_1,1_* refers to mortality under ambient conditions, G*_2,2_* refers to mortality under acidified conditions, and the off‐diagonal elements represent the genetic covariance. Changes in average phenotype can be calculated as Δz=Gβ, where *β* is the vector of selection gradients (Lande, 1979). Supposing that changes in seawater chemistry result in a modest amount of selection on larval mortality (e.g., *β* = $\begin{array}{c} -\text{0.1}\\ \text{0} \end{array}$; note that selection in the current generation acts on mortality under ambient conditions only), then larval mortality rate under current conditions is expected to change by 2.48 × 10^−4^ × −0.1 = −2.48 × 10^−5^ per day on average, and through the correlated response to selection in the current generation, mortality rates under acidified conditions would change by 2.10 × 10^−4^ × −0.1 = −2.10 × 10^−5^ per day. Thus, by changing the relative frequencies of phenotypes and genotypes within the population, selection in the current generation can make the population more tolerant of future, more acidified conditions, even though the population may not experience the projected conditions for several‐to‐many generations. As the environment transitions toward a more acidified state and directional selection occurs, correlated responses will be a key part of the adaptation process. The magnitude of correlated genetic responses and the cumulative changes in allele frequencies over time will thus be major determinants of evolutionary rate (Etterson & Shaw, 2001; Via & Lande, 1985). In order to accurately assess the potential to evolve in response to ocean acidification, it is therefore imperative to study both heritabilities and genetic correlations of fitness‐related traits in ambient and acidified seawater environments.

Finally, it should be emphasized that measures of heritabilities and genetic correlations across a range of OA conditions evaluate only the *potential *to evolve. Although measuring these inheritance parameters will be an essential first step, rates of evolution will also depend on selection, which may be a complex and nonlinear process (Chevin, Lande, & Mace, 2010; Lande & Shannon, 1996). Understanding how changes in ocean chemistry will affect the form and rate of selection on larval phenotypes should be a priority for future studies of ocean acidification. If both selection and genetic capacity to evolve can be measured, then one can project evolutionary responses and their effects on population dynamics. Such information will be essential for understanding the long‐term effects of ocean acidification on marine populations (Munday, Warner, Monro, Pandolfi, & Marshall, 2013; Reusch, 2014; Sunday et al., 2014).

## CONFLICT OF INTEREST

None declared.

## DATA ARCHIVING STATEMENT

Data available from the Dryad Digital Repository: https://doi.org/10.5061/dryad.kf0h22h
