# Supplementary material for: Can larvae of a marine fish adapt to ocean acidification? Evaluating the evolutionary potential of California Grunion (Leuresthes tenuis)
Source: Evol Appl. 2018 Dec 31;12(3):560–71. doi: 10.1111/eva.12739 (PMC6383732; doi:10.1111/eva.12739)
Supplement: Supplementary file 1 [file EVA-12-560-s001.docx]

**Appendix S1. Effects of seawater *p*CO_2_ and pH on mortality rates of larval fishes**

To summarize the effects of changes in seawater *p*CO_2_ and pH on the mortality rates of larval fishes, we conducted a review and analysis of published results. Using Google Scholar and Web of Science search engines, we searched for experimental studies that exposed larvae to ocean acidification conditions and measured mortality rates. For each study we found, we examined the reference lists to find similar studies, and we searched the lists of studies that cited each article to find other, newer studies in this field. In total, we were able to summarize data for 124 replicates, 46 experiments, and 13 different species.

| Data on survival were obtained from each study and converted to measures of instantaneous mortality rates (per day). To account for natural differences in overall mortality rates among species and to isolate the component of mortality due to changes in pH and *p*CO_2_, we calculated “added mortality” by subtracting the average mortality rate at ambient conditions from all replicates in the corresponding experiment. To estimate the average effect of OA conditions on mortality, and to account for the fact that these relationships will vary among species, we used a linear mixed effects model to describe the relationship between *p*CO_2_ and added mortality. We ran similar models to describe the relationship between pH and added mortality. Specifically, we used a model in which the slopes and intercepts were treated as random effects and allowed to vary among species. Models were fit using the lme4 package in R (Bates et al. 2015). In addition to estimating the average effect of *p*CO_2_/pH on mortality rates, we also used the Best Linear Unbiased Predictors for each random effect to describe the effects of *p*CO_2_/pH on mortality rates for each species. From this overall model, the slopes provide a measure of OA tolerance for that species. Values near zero indicate tolerance whereas large, negative values indicate a high degree of sensitivity. Specifically, the slope values describe how much a unit change in *p*CO_2_/pH affects instantaneous mortality rate. Data are summarized in Table S1. We note that although species’ responses varied in magnitude, no one species had an overwhelming influence on the average relationship between pH/*p*CO_2_ and mortality rates.  Table S1. A summary of the relationships between pH/*p*CO_2_ and mortality rates of marine fish larvae. Slope values are the Best Linear Unbiased Predictors for the relationships between pH/*p*CO_2_ and daily mortality rate for each species. |
| --- |
|  |

**Appendix S2. Summary of seawater chemistry parameters**

Two, recirculating seawater systems were constructed to manipulate the carbonate chemistry of seawater and rear larval fish within the lab (Fig. S1). Systems consisted of 12 larval rearing tanks (each 6.6 L in volume) that drained into a common sump where water was filtered and aerated. In the ocean acidification treatment, both air and carbon dioxide were bubbled into the sump. In the ambient treatment, air was bubbled into the sump. Otherwise the treatments were identical.

| 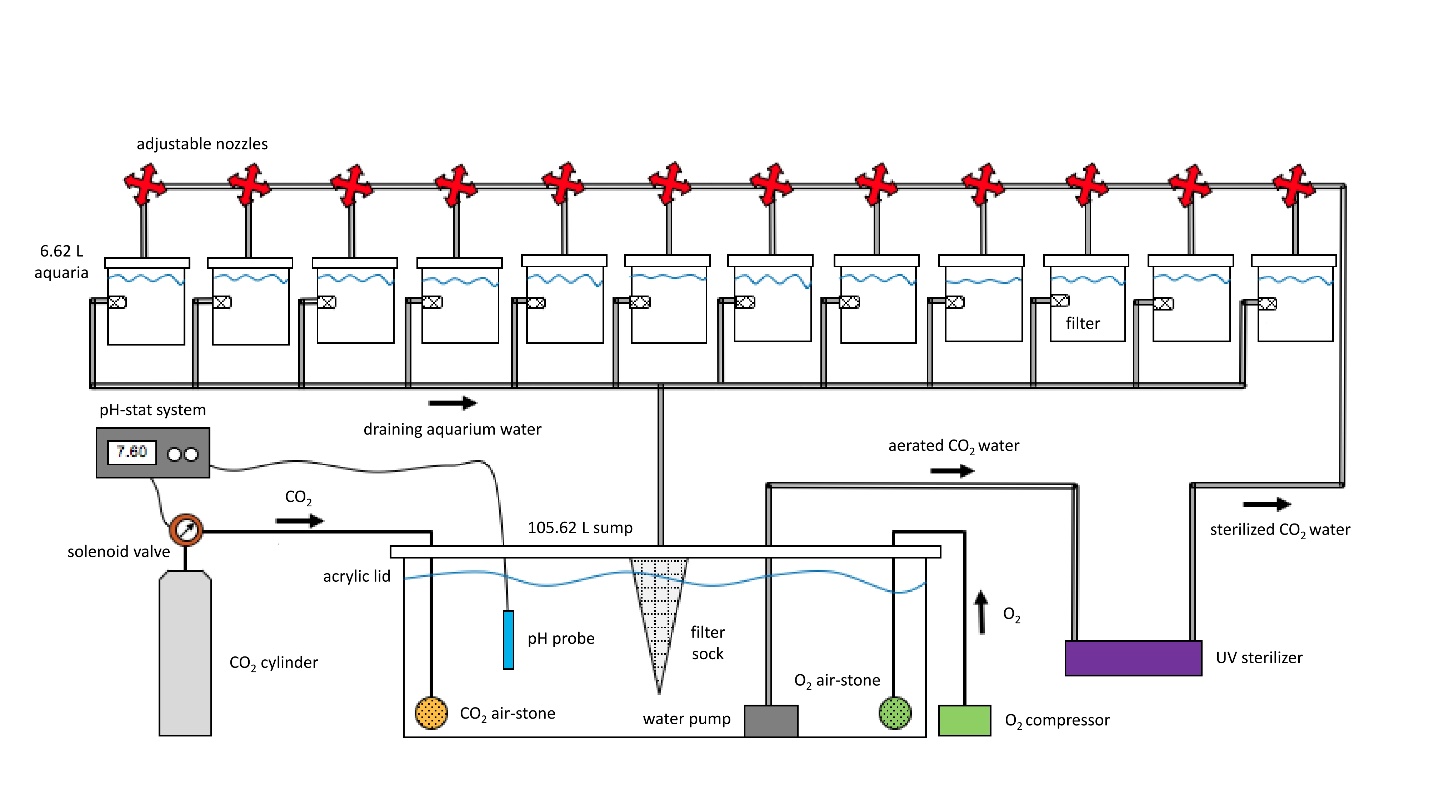 |
| --- |
| Figure S1. An illustration of the seawater system used in this study. |

Table S2 summarizes the water chemistry and carbonate parameters that were measured throughout the experiment. Note that our experiment was replicated in 16 blocks over time and that we replaced the water in between blocks. Experimental systems were replenished with seawater collected from local, nearshore environments, and in all cases the same collection of seawater was used to replace both the ambient and ocean acidification (OA) treatments. Within blocks, the salinity, temperature, and total alkalinity did not differ between the ambient and OA treatments. However, total alkalinity varied substantially among blocks, reflecting the natural variation in alkalinity levels experienced over time along our coast. Even in the presence of among-block variability, our analysis was hierarchical in that we examined genetic variation by comparing responses of various types of relatives and unrelated individuals within each block. This allowed us to parse out genetic variation in mortality and growth while also accounting for overall variation among blocks.

| Table S2. Summaries of water chemistry parameters. Note that pH, salinity, temperature and total alkalinity were measured during the experiment. All other attributes were calculated with the program CO2SYS (Pierrot et al., 2006). |
| --- |
| **** |

**References**

Bates, D., Maechler, M., Bolker, B., & Walker, S. (2015). Fitting linear mixed-effects models using lme4. *Journal of Statistical Software*, 67, 1-48.

Pierrot, D.E.L., & Wallace, D.W.R. (2006). MS Excel program developed for CO_2_ system calculations. ORNL/CDIAC-105a. Carbon Dioxide Information Analysis Center, Oak Ridge National Laboratory, US Department of Energy, Oak Ridge, Tennessee. doi: 10.3334/CDIAC/otg. CO2SYS_XLS_CDIAC105a
